# Comparison of Active Drug Concentrations in the Pulmonary Epithelial Lining Fluid and Interstitial Fluid of Calves Injected with Enrofloxacin, Florfenicol, Ceftiofur, or Tulathromycin

**DOI:** 10.1371/journal.pone.0149100

**Published:** 2016-02-12

**Authors:** Derek M. Foster, Luke G. Martin, Mark G. Papich

**Affiliations:** 1 Department of Population Health and Pathobiology, College of Veterinary Medicine, NC State University, Raleigh, NC, United States of America; 2 Department of Molecular and Biomedical Sciences, College of Veterinary Medicine, NC State University, Raleigh, NC, United States of America; The Ohio State University, UNITED STATES

## Abstract

Bacterial pneumonia is the most common reason for parenteral antimicrobial administration to beef cattle in the United States. Yet there is little information describing the antimicrobial concentrations at the site of action. The objective of this study was to compare the active drug concentrations in the pulmonary epithelial lining fluid and interstitial fluid of four antimicrobials commonly used in cattle. After injection, plasma, interstitial fluid, and pulmonary epithelial lining fluid concentrations and protein binding were measured to determine the plasma pharmacokinetics of each drug. A cross-over design with six calves per drug was used. Following sample collection and drug analysis, pharmacokinetic calculations were performed. For enrofloxacin and metabolite ciprofloxacin, the interstitial fluid concentration was 52% and 78% of the plasma concentration, while pulmonary fluid concentrations was 24% and 40% of the plasma concentration, respectively. The pulmonary concentrations (enrofloxacin + ciprofloxacin combined) exceeded the MIC_90_ of 0.06 μg/mL at 48 hours after administration. For florfenicol, the interstitial fluid concentration was almost 98% of the plasma concentration, and the pulmonary concentrations were over 200% of the plasma concentrations, exceeding the breakpoint (≤ 2 μg/mL), and the MIC_90_ for *Mannheimia haemolytica* (1.0 μg/mL) for the duration of the study. For ceftiofur, penetration to the interstitial fluid was only 5% of the plasma concentration. Pulmonary epithelial lining fluid concentration represented 40% of the plasma concentration. Airway concentrations exceeded the MIC breakpoint for susceptible respiratory pathogens (≤ 2 μg/mL) for a short time at 48 hours after administration. The plasma and interstitial fluid concentrations of tulathromcyin were lower than the concentrations in pulmonary fluid throughout the study. The bronchial concentrations were higher than the plasma or interstitial concentrations, with over 900% penetration to the airways. Despite high diffusion into the bronchi, the tulathromycin concentrations achieved were lower than the MIC of susceptible bacteria at most time points.

## Introduction

Measurement of antimicrobial concentrations at the site of infection is crucial for prediction of antimicrobial efficacy. Yet, traditional measures of antibiotic concentrations including plasma, tissue cages, and homogenized lung tissue appear to be poor predictors of drug concentrations in the pulmonary epithelial lining fluid (PELF), the initial site of bacterial colonization [1–5]. To better quantify antimicrobial concentrations in the airways of calves without euthanasia, two techniques have been developed. The first [3–5] consists of passing a guarded swab through either the nose or mouth, down the trachea to a bronchus. The swab is then passed into the bronchus, and PELF is absorbed in the swab. PELF is then extracted from the swab allowing for measurement of drug concentration. The second approach [1–2] is similar to a diagnostic bronchoalveolar lavage (BAL) in which a catheter is passed through the nostril, down the trachea and lodged into a bronchus. A balloon on the end of the catheter is inflated to lodge the catheter in place, and sterile saline is lavaged into the lung, and quickly aspirated. The aspirate can be assayed for the drug of interest. Advantages of the swab technique include direct measurement of drug concentrations without the need to correct for dilution and the ability to repeatedly sample the same animal without concern for the volume of fluid required for sampling. This swab technique is now generally accepted as a preferred method in order to avoid the methodological difficulties associated with BAL methods [6–7]. According to Kiem & Schentag [7], the direct microsampling technique “may offer an overall better correlation with microbiological outcomes.” Further, it is widely accepted that collection of protein-free ultrafiltrate from tissues is the most accurate and reliable measure of free (microbiologically active) drug concentration at the tissue site [8–10], and this *in vivo* ultrafiltration technique has been shown to be effective and easily adapted to large animals [11–12].

For studies of antibiotics directed at treating bovine respiratory disease (BRD) it is not known what matrix should be used to measure drug concentration to predict clinical outcome and to make predictions based on drug concentrations in relation to the minimum inhibitory concentration (MIC) and pharmacokinetic-pharmacodynamic (PK-PD) modeling techniques. The objective of this study was to investigate pharmacokinetics of four antimicrobials administered to calves for treatment and control of BRD. These four drugs (enrofloxacin, florfenicol, ceftofur crystalline-free acid, and tulathromycin) are commonly used in cattle and represent four distinct classes of antimicrobials. As these drugs differ in protein binding and lipophilicity, the objective of the study was to examine the impact of these properties on diffusion into the PELF. In this study, each calf received a single administration per label instructions. Following injection, interstitial fluid, PELF fluid, and plasma were collected at regular intervals to characterize the disposition of each antimicrobial and compare drug concentrations in each matrix.

## Materials and Methods

### Experimental animals

Twelve 6 month old Holstein steer calves were obtained from the Dairy Education Unit of North Carolina State University. The protocol was approved by the Institutional Animal Care and Use Committee (IACUC) of North Carolina State University prior to initiation of the study. The calves were accurately weighed and examined to ensure that they were healthy prior to the study. The calves were housed in pairs at the Laboratory Animal Resources facility at the NC State College of Veterinary Medicine for the duration of the study.

### Allocation of Animals

Random assignment were made to each drug treatment group with 6 calves in each group. All drugs were administered in the neck (except ceftiofur) according to label instructions. Group 1 received enrofloxacin (Baytril 100, Bayer HealthCare LLC, Animal Health Division, Shawnee Mission, KS, USA) at a dose of 7.5 mg/kg of body weight once subcutaneously. Group 2 received florfenicol + flunixin meglumine (Resflor Gold^®^, Merck Animal Health, Whitehouse Station, NJ) at a dose of 40 mg/kg of body weight once subcutaneously. Group 3 received ceftiofur crystalline free acid suspension (Excede^®^, Zoetis Animal Health, Florham Park, NJ, USA) at a dose of 6.6 mg/kg, once as single subcutaneous injection at the base of the posterior aspect of the ear. Group 4 received tulathromycin (Draxxin^®^, Zoetis Animal Health, Florham Park, NJ, USA) at a dose of 2.5 mg/kg of body weight once subcutaneously. The calves were allocated into two period semi-crossover design. Six calves received enrofloxacin, and following the sampling period and a 7 day washout period, they received florfenicol. The week following the conclusion of the florfenicol sampling, a second set of 6 steers were administered ceftiofur. Following the sampling period and a 7 day washout period, those 6 steers received tulathromycin.

### Blood Collection

Prior to drug administration a 14 gauge, 13 cm jugular catheter (Intracath^®^, Becton Dickinson, Franklin Lakes, NJ) was inserted in the jugular vein for the purpose of collecting blood samples. Blood samples were collected at time 0, and at appropriate intervals for optimum pharmacokinetic modeling, accounting for 90% of drug elimination. Plasma samples were collected at time 0, 5, 10, 15, 30, 45, 60, 90, 120 min, and 4, 6, 8, 12, 24, 30, and 48 hr after enrofloxacin administration and every 24 hours for up to 288 hours for the other drugs.

### Bronchial Fluid Collection

Bronchial swabs were collected at 6, 24, 30, and 48 hr after administration of enrofloxacin, and then every 48 hr for up to 288 hr for the other drugs. A square of cotton gauze was grasped by 3 meter endoscopic biopsy forceps (Endoscopic Support Services, Brewster, NY) and fed into a flexible orogastric tube (9.5 mm in diameter, 2 m long, Santa Cruz Animal Health, Dallas, TX) so that the gauze obstructed the end of the tube. The flexible tube with the preplaced biopsy forceps with gauze was passed through a nostril and down the trachea until it lodged in a bronchus. The cotton gauze preventing nasal and tracheal contamination was then removed. The endoscopic biopsy forceps were used to grasp a half circle of absorbent filter paper (25 mm Glass Microfiber Filters, Whatman/GE Healthcare Life Sciences, Pittsburg, PA) which was passed through the flexible tube until it extended 6cm beyond the tube into a bronchus. The filter paper was allowed to contact the bronchus for 15 seconds, then retracted into the flexible tube to prevent contamination during withdrawal through the trachea and nostril. The absorbent filter paper was released from the jaws of the biopsy forceps into a sterile clean glass tube. An animation of this collection method can be viewed in the supplementary figure (S1 Fig). The weight of the sample paper was recorded (by subtracting a pre-sample tare weight). The weight of the filter paper was used to determine the volume of PELF absorbed into the absorbent paper.

### Collection of ISF

The steers were fitted with an in-vivo ultrafiltration sampling kit (BAS Bioanalytical Systems, W. Lafayette, IN) as previously described [11–12]. The ultrafiltration probe was placed subcutaneously over the withers. The probe contains 3 semi-permeable loops connected to a non-permeable tube extending to the exterior of the animal and attached to an evacuated blood collection tube with no additives. The evacuated tube provides the negative pressure for fluid collection through the small pores in the loop membrane. The membrane in the loop consists of pores allowing water, electrolytes, and low molecular weight molecules (less than 30,000 Daltons) to pass and excludes the passage of protein, protein-bound drugs, and other large molecular weight compounds. The interstitial fluid was collected at time 0 and at appropriate intervals for each drug which accounted for at approximately 3 drug half-lives after administration. The samples were collected at Time 0, 2, 4, 6, 12, 24, 30, and 48 hr after administration of enrofloxacin, and then every 24 hr for up to 288 hr for the other drugs. The fluid collected was immediately frozen at -80°C until analysis.

### Plasma Protein Binding

Protein binding was determined with a micorcentrifugation system (Centrifree^™^ Micropartition system, Amicon, Beverly, MA). Pooled plasma was collected from six healthy calves. Aliquots of calf plasma were fortified (spiked) with the drug of interest to make three levels at the low, medium and high concentrations anticipated in the plasma after drug administration. In addition to adding the parent drug ceftiofur to samples, we also measured the protein binding of the main metabolite of ceftiofur, desfuroylceftiofur, by collecting incurred samples from calves, and processing these in the same manner as spiked samples. Replicates of three samples at each concentration were prepared and incubated for 30 minutes. Each spiked plasma sample (or incurred samples from 3 calves for measurement of the ceftiofur metabolite) was added to a microcentrifugation system. These specially designed systems are disposable and rapidly and efficiently separate free from protein-bound drug in plasma or other biological fluids. After adding 1.0 mL spiked plasma to the microcentrifugation system, the sample tube was centrifuged at 1,000 x g for 10 minutes. A protein free ultrafiltrate (approximately 30% of the sample volume) was obtained in the reservoir of the system. The recovered ultrafiltrate was analyzed directly by high performance liquid chromatography (HPLC). Concentration of the unbound fraction was determined from a calibration curve of fortified blank ultrafiltrate solution. Protein binding of each drug was calculated according to the following formula:

$$\%\;Protein\;Binding=\frac{\left[total-unbound\right]}{total}*\;100$$

### Drug Analysis

Plasma and bronchial fluid samples were analyzed by reverse-phase HPLC with UV detection using methods previously published by our laboratory [11, 13–14]. Ceftiofur and active metabolites were analyzed using a method adapted from Jaglan et al [15]. Drug concentrations of tulathromycin were measured using a liquid chromatography-mass spectrometry method (LC-MS) developed in the College’s MS facility. The method was adapted from a previously published technique [16].

Drug concentrations were extracted from plasma. Interstitial fluid was analyzed directly, without extraction. The swabs containing PELF were extracted with a solvent, and processed in a similar manner. The solvent used for each drug was: a solution of 70% distilled water/30% acetonitrile for florfenicol, 87% distilled water/13% acetonitrile for ceftiofur and metabolites, 85% trifluoroacetic acid (0.1%)/15% methanol for enrofloxacin/ciprofloxacin, and 90% 0.02 M ammonium acetate buffer/10% acetonitrile for tulathromycin. Extraction from the swabs was near 100% recovery. If recovery of drug was not 100%, the result was corrected for the fraction extracted.

### Pharmacokinetic Analysis

The drug concentrations were analyzed using standard pharmacokinetic methods to determine the drug disposition for each drug in each calf. A computer program (Phoenix^®^ WinNonlin, Version 6.2 Certara, U.S.A. Inc. Princeton, NJ) was used to determine pharmacokinetic parameters as well as to derive statistical values. Both compartmental and non-compartmental analysis was used. The nature of the data determined what approach was used. If data could be adequately described using compartmental methods, these were reported in the results. If a compartmental pharmacokinetic model could not adequately be fit to the data (determined by goodness-of-fit criteria) a non-compartmental method was reported.

Pharmacokinetic methods used in our laboratory follow the guidelines and calculations described by Gibaldi and Perrier [17]. Among the data presented, mean plasma and tissue fluid concentrations, and mean values for peak concentration (Cmax_)_, time to peak concentration (Tmax_)_, terminal half-life (T½) and area under the curve (AUC) are reported. An assessment of the drug distribution to the ISF and PELF was analyzed by measuring AUC ratios from each site compared to plasma concentration. A *penetration factor* was calculated from the AUC [7] which represents the ratio of the drug concentration in each tissue fluid compared to plasma drug concentration.

## Results

There were no adverse reactions to any drugs, except obvious discomfort from the injection of florfenicol + flunixin meglumine presumably caused by pain at the injection site. One calf died unexpectedly several days after the enrofloxacin administration. The calf was necropsied, and it was determined that the calf died of juvenile lymphoma unrelated to the study. This calf was replaced to complete the study.

### Pharmacokinetic Analysis

The drug concentrations were analyzed using both compartmental and non-compartmental pharmacokinetic methods to determine the drug disposition for each drug and each tissue site in each calf. In some tissues and for some drugs, a compartmental approach was used because a model could be easily fitted to the data because of rich sampling points. In other tissue fluids and for some drugs, there were not enough data points to fit a model to the curve and a non-compartmental approach was used.

**Fig 1:**
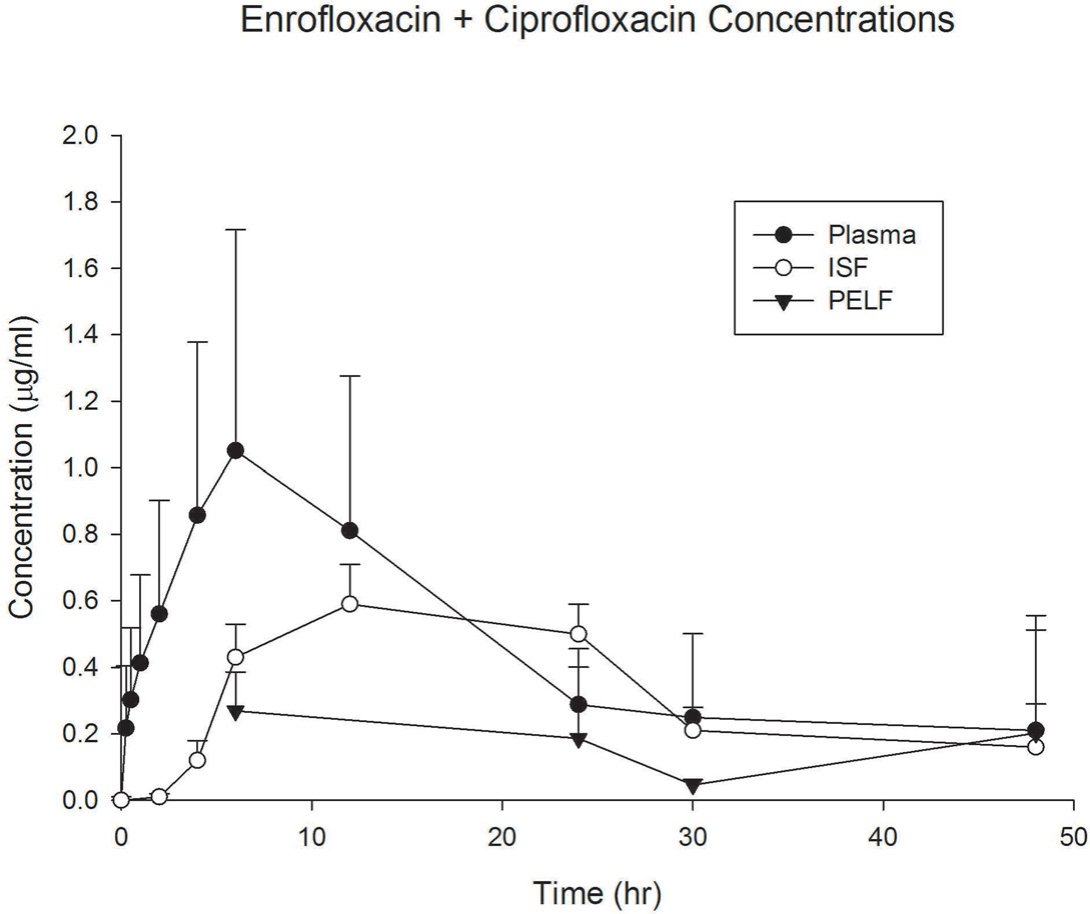
Enrofloxacin + Ciprofloxacin Concentrations. Plasma, interstitial fluid (ISF), and pulmonary epithelial lining fluid (PELF) concentrations of enrofloxacin + ciprofloxacin (concentrations summed) in cattle (n = 6) after administration of enrofloxacin (7.5 mg/kg s.c.). Data are presented as mean +/- standard deviation.

Terminal T½ for enrofloxacin and its metabolite ciprofloxacin in plasma was 9.23 hr and 14.7 hrs, respectively (Table 1). This was slightly longer than the half-life from a previous study in other calves using a higher dose [11]. Protein binding was approximately 46% for enrofloxacin and 19% for ciprofloxacin, representing a *fu* of 0.54 and 0.81, respectively (Table 2). The ISF concentration for enrofloxacin and ciprofloxacin was 52% and 78% of the plasma concentration (Table 3, Fig 1), respectively, agreeing closely with the expected concentrations predicted from the fraction unbound (*fu*), as one expects the unbound plasma concentration in plasma to be in equilibrium with the interstitial fluid concentration. PELF concentrations were 24% and 40% of the plasma concentration for enrofloxacin and ciprofloxacin, respectively (Table 4, Fig 1). The PELF concentrations (enrofloxacin + ciprofloxacin combined) exceeded the reported MIC_90_ of 0.06 μg/mL at 48 hours after administration. The PELF AUC concentrations were 5.72 μg hr/mL. Using a ratio of AUC/MIC > 100, this indicates that AUC in PELF is close to achieving the target for MIC values < 0.06 μg/mL. Using the same target AUC/MIC ratio of 100, the AUC for the ISF of 12.85 μg hr/mL exceeds the target for MIC values of 0.06 μg/mL by a factor of more than 2-fold.

**Table 1:** Plasma Pharmacokinetics of Enrofloxacin and Ciprofloxacin after Administration of 7.5 mg/kg of Enrofloxacin to Calves.

		Enrofloxacin		Ciprofloxacin		Enrofloxacin + Ciprofloxacin	
Parameter	Units	Mean	CV%	Mean	CV%	Mean	CV%
AUC	hr*ug/mL	17.79	61.43	8.04	30.30	25.84	49.93
Cmax	ug/mL	0.89	47.90	0.30	42.52	1.19	45.11
K_01_	1/hr	0.98	136.05	0.50	49.42		
K_01_ T½	hr	1.67	48.63	1.78	53.09		
K_10_	1/hr	0.08	27.18	0.05	24.06		
K_10_ T½	hr	9.23	25.95	14.71	23.65		
Tmax	hr	4.65	38.65	5.72	27.49		

AUC, Area-under-the-curve; Cmax, peak (maximum) concentration; K_01_, absorption rate and associated half-life T½; K_10_, elimination rate (terminal rate) and associated half-life T½; Tmax, time to peak concentration. Blank spaces indicate that it is inappropriate to combine these parameters for both drugs.

**Table 2:** Effect of Lipophilicity and Protein Binding on Penetration into Interstitial Fluid (ISF) and Pulmonary Epithelial Lining Fluid (PELF). LogD = log of the partition coefficient at pH 7.4. A higher LogD value indicates a greater lipophilicity (Source: ChemSpider, www.chemspider.com). Fraction unbound is the mean from three replicates. *Source for Protein Binding Data*: Florfenicol, ceftiofur, from this study. Enrofloxacin, ciprofloxacin from Davis, Foster & Papich 2007; tulathromycin, Pfizer Freedom of Information (FOI) Summary NADA 141–244, 2005 for Draxxin injectable solution (tulathromycin).

			Percent Penetration	
	LogD	Fraction Unbound (*fu*)	ISF	PELF
Florfenicol	-0.12	0.95–0.99	98%	261%
Enrofloxacin	0.66	0.54	52%	24%
Ciprofloxacin	-1.26	0.81	78%	40%
Ceftiofur	-1.72	0.42	-	-
Ceftiofur metabolite *	-	0.067	5%	40%
Tulathromycin	-1.06	0.53–0.68	44%	905%

* Data for ceftiofur metabolite was derived from incurred samples (after calves converted ceftiofur to main metabolite).

**Table 3:** Interstitial Fluid (ISF) Pharmacokinetics of Enrofloxacin and Ciprofloxacin after Administration of 7.5 mg/kg of Enrofloxacin to Calves.

		Enrofloxacin		Ciprofloxacin		Enrofloxacin + Ciprofloxacin	
Parameter	Units	Mean	CV%	Mean	CV%	Mean	CV%
AUC (0 to Cn)	h*ug/mL	6.98	47.88	4.74	49.88	11.72	47.14
AUC (0 to infinity)	h*ug/mL	8.99	21.11	7.08	21.00	12.85	59.36
Cmax	ug/mL	0.32	47.73	0.19	39.22	0.51	41.11
Ke T ½	h	10.08	15.28	16.25	25.81		
Ke	1/h	0.07	16.68	0.04	25.55		
MRT	h	19.66	12.73	29.26	15.24		
Tmax	h	15.60	51.60	18.00	47.14		
Penetration factor	%	52	46.19	78	8.99	59	34.08

AUC, Area-under-the-curve, from zero to the last time point (Cn), and from 0 to infinity; Cmax, peak (maximum) concentration; Tmax, time to peak concentration; Penetration factor, ratio of AUC values of tissue site: plasma, expressed as percent; Ke, elimination (terminal) rate, and associated half-life, T½. Blank spaces indicate that there was not enough data to estimate this parameter.

**Table 4:** Pulmonary Epithelial Lining Fluid (PELF) Pharmacokinetics of Enrofloxacin and Ciprofloxacin after Administration of 7.5 mg/kg of Enrofloxacin to Calves.

		Enrofloxacin		Ciprofloxacin		Enrofloxacin + Ciprofloxacin	
Parameter	Units	Mean	CV%	Mean	CV%	Mean	CV%
AUC (0 to Cn)	h*ug/mL	3.53	33.1	1.57	24.3	5.10	26.5
AUC (0 to infinity) (0 to infinity)	h*ug/mL	4.03	32.1	3.05	53.2	5.72	68.6
Cmax	ug/mL	0.19	40.8	0.06	30.9	0.25	34.9
Ke T ½	h	12.76	53.7	43.58	66.2		
Ke	1/h	0.07	51.4	0.02	40.0		
MRT	h	15.83	16.5	65.54	61.3		
Tmax	h	6.00	0.0	19.00	88.0		
Penetration factor	%	24	38.6	40	64.5	27	50.3

AUC, Area-under-the-curve, from zero to the last time point (Cn), and from 0 to infinity; Cmax, peak (maximum) concentration; Tmax, time to peak concentration; Penetration factor, ratio of AUC values of tissue site: plasma; Ke, elimination (terminal) rate, and associated half-life, T½. Blank spaces indicate that there was not enough data to estimate this parameter.

**Fig 2:**
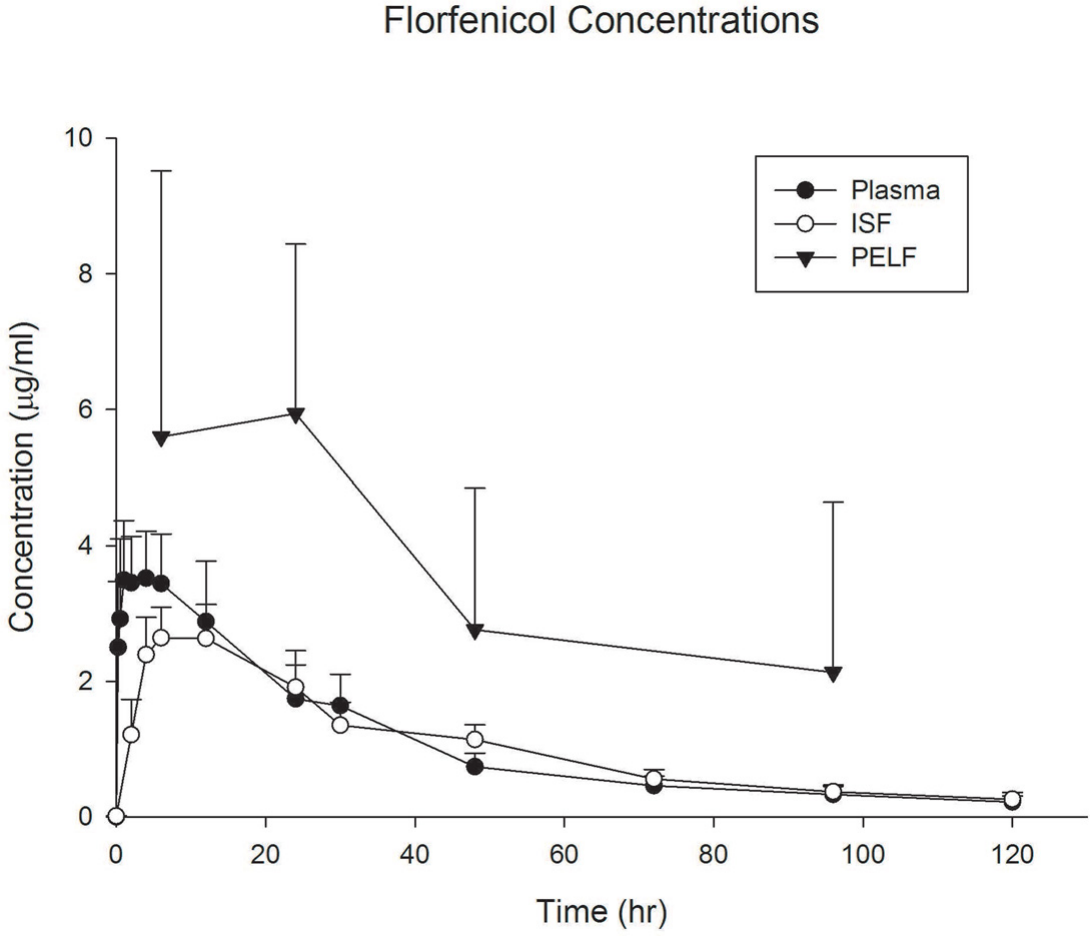
Florfenicol Concentrations. Plasma, interstitial fluid (ISF), and pulmonary epithelial lining fluid (PELF) concentrations of florfenicol in cattle (n = 6) after administration of florfenicol (40 mg/kg s.c.). Data are presented as mean +/- standard deviation.

Florfenicol protein binding was only 5% at the high concentration and was negligible at the low concentrations, representing a *fu* of essentially 1.0 (Table 2). The terminal T½ was approximately 28 hours, agreeing with label information. The ISF concentration was almost 98% of the plasma concentration, as expected from the low protein binding (high *fu*). The PELF concentrations exceeded both plasma and ISF concentrations and were over 200% of the plasma concentrations (Table 5, Fig 2). The PELF concentrations exceeded the breakpoint of ≤ 2 μg/mL, and the MIC90 for *Mannheimia haemolytica* (1.0 μg/mL) for BRD pathogens for the duration of the study.

**Table 5:** Plasma, Insterstitial Fluid (ISF), and Pulmonary Epithelial Lining Fluid (PELF) Pharmacokinetics after Administration of 40 mg/kg of Florfenicol to calves.

		Compartmental Analysis				Non-Compartmental Analysis			
		Plasma		ISF		PELF			
Parameter	Units	Mean	CV%	Mean	CV%	Parameter	Units	Mean	CV%
AUC	hr*ug/mL	142.90	22.15	137.18	16.24	AUC	hr*ug/mL	342.27	43.56
Cmax	ug/mL	3.42	23.72	2.56	19.56	C_LAST_	ug/mL	2.13	117.89
K_01_	1/hr	5.11	44.52	0.36	25.74	Cmax	ug/mL	7.52	44.85
K_01_ T½	hr	0.16	47.34	2.02	26.70	Ke T½	hr	32.04	4.66
K_10_	1/hr	0.02	17.18	0.02	12.00	Ke	1/hr	0.02	4.66
K_10_ T½	hr	28.44	15.78	30.99	11.49				
Tmax	hr	1.19	39.99	8.46	21.01				
Penetration Factor		%		97	11.71	Penetration Factor		261	33.03

AUC, Area-under-the-curve; Cmax, peak (maximum) concentration; K_01_, absorption rate and associated half-life T½; K_10_, elimination rate (terminal rate) and associated half-life T½; Tmax, time to peak concentration; Penetration factor, ratio of AUC values of tissue site: plasma; Ke, elimination (terminal) rate, and associated half-life, T½; C_LAST_, concentration at last time point measured.

**Fig 3:**
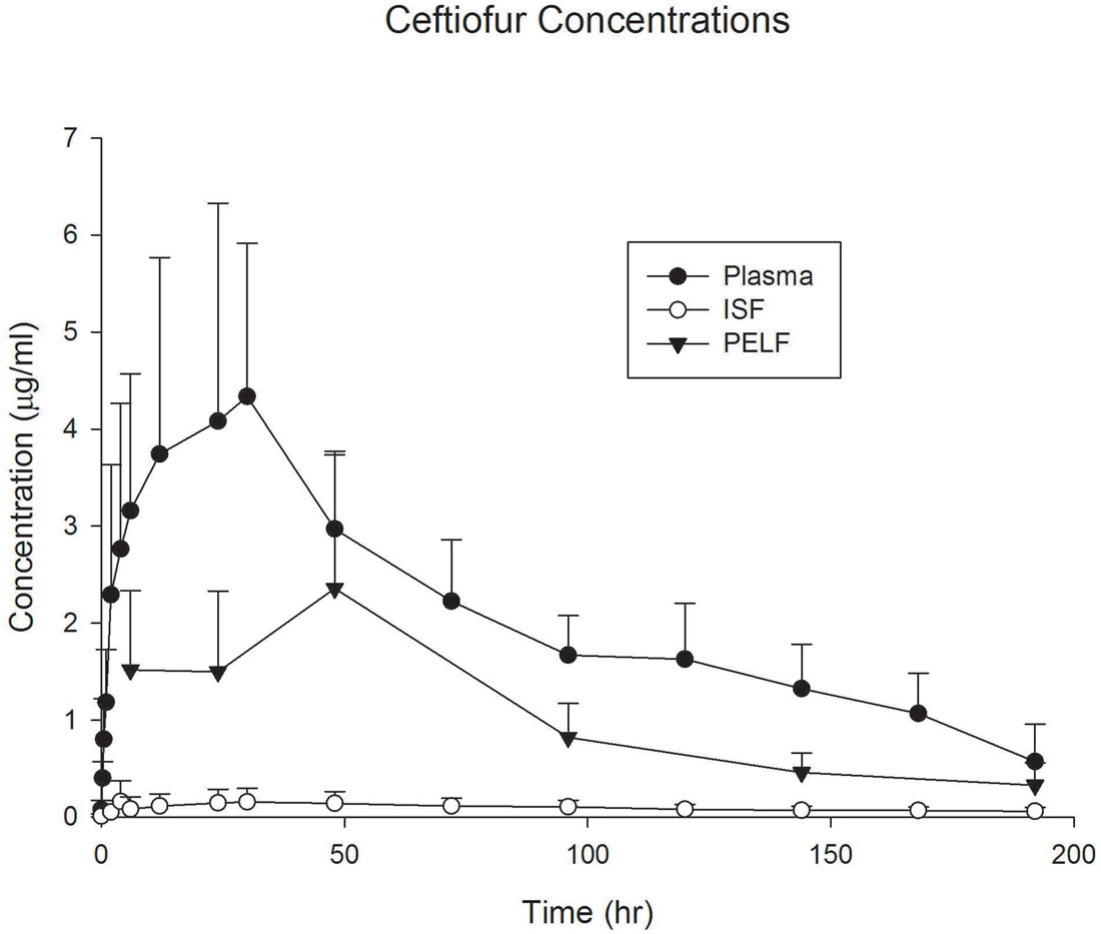
Ceftiofur Concentrations. Plasma, interstitial fluid (ISF), and pulmonary epithelial lining fluid (PELF) concentrations of ceftiofur and its metabolites in cattle (n = 6) after administration of ceftiofur crystalline free acid (6.6 mg/kg s.c. at the base of the ear). Data are presented as mean +/- standard deviation.

Ceftiofur protein binding was approximately 52% and 63%, respectively for the high and low concentration of the parent drug studied, representing an average fraction unbound (*fu*) of 0.42 (Table 2). The protein binding from the main ceftiofur metabolite, desfuroylceftiofur, from incurred samples obtained from these calves was 93.3% at concentrations ranging from 2.75–6.0 μg/mL, representing a *fu* of 0.067. Terminal plasma half-life (T½) for this ceftiofur slow-release formulation, as expected, was over 103 hours. The ISF concentrations were lower than anticipated when considering only the ceftiofur parent drug with penetration to the ISF only 5% of the plasma concentration. However, when considering the protein binding of the main metabolite desfuroylceftiofur (93.3%, Table 2), the penetration into ISF is in agreement with what is anticipated. Penetration of ceftiofur and associated metabolites to the PELF was higher than anticipated and represented 40% of the plasma concentration (Table 6, Fig 3). Concentrations in PELF exceeded the MIC breakpoint listed by CLSI for susceptible BRD pathogens (2 μg/mL) for only a short time at approximately 48 hours after administration. Because of low penetration, concentrations in the ISF never reached a level above the MIC_90_ for BRD pathogens at any time point.

**Table 6:** Plasma, Insterstitial Fluid (ISF), and Pulmonary Epithelial Lining Fluid (PELF) Pharmacokinetics of Ceftiofur and Ceftiofur Derivatives after Administration of 6.6 mg/kg of Ceftiofur Crystalline Free Acid to calves.

		Plasma		ISF		PELF	
Parameter	Units	Mean	CV%	Mean	CV%	Mean	CV%
AUC	hr*ug/mL	554.74	17.94	26.04	49.57	211.94	23.23
Cmax	ug/mL	4.26	42.33	0.20	59.79	2.09	53.60
K_01_	1/hr	0.334	42.764	0.101	71.721	0.167	144.827
K_01_ T½	hr	2.51	55.02	19.39	141.27	9.95	55.15
K_10_	1/hr	0.009	57.384	0.013	42.162	0.015	47.450
K_10_ T½	hr	103.65	65.87	58.97	32.03	58.74	58.94
Tmax	hr	13.14	53.24	64.71	107.71	31.26	45.24
Tissue Penetration (%)				5	59.96	40	37.94

AUC, Area-under-the-curve; Cmax, peak (maximum) concentration; K_01_, absorption rate and associated half-life T½; K_10_, elimination rate (terminal rate) and associated half-life T½; Tmax, time to peak concentration; Penetration factor, ratio of AUC values of tissue site: plasma.

**Fig 4:**
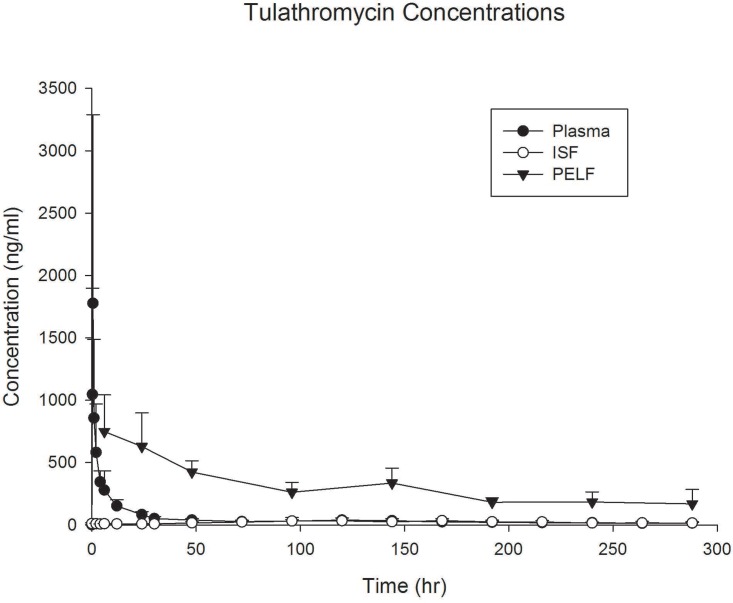
Tulathromycin Concentrations. Plasma, interstitial fluid (ISF), and pulmonary epithelial lining fluid (PELF) concentrations of tulathromycin in cattle (n = 6) after administration of tulathromycin (2.5 mg/kg s.c.). Data are presented as mean +/- standard deviation.

The tulathromycin results are shown in Table 7 and Fig 4. The figure and table show concentrations in PELF greatly exceeded the plasma drug concentrations throughout the collection period. The data in Table 6 shows that based on tissue/plasma concentration ratio (ratio of AUC values), PELF exposure was over 9 x higher than plasma (CV 45%). Protein binding closely predicted ISF concentrations, while PELF concentrations greatly exceeded the plasma and ISF concentrations (Table 2).

**Table 7:** Plasma, Insterstitial Fluid (ISF), and Pulmonary Epithelial Lining Fluid (PELF) Pharmacokinetics after Administration of 2.5 mg/kg of Tulathromycin to Calves.

		Plasma		ISF		PELF	
Parameter	Units	Mean	CV%	Mean	CV%	Mean	CV%
AUC % extrapolated	%	8.25	37.96	15.88	86.29	24.9	54.1
AUC (0 to infinity)	hr*μg/mL	14.48	28.61	8.60	7.50	11.99	27.6
AUC (0 to Cn)	hr*μg/mL	13.23	26.62	5.54	39.75	8.76	21.7
Cmax	μg/mL	1.82	82.28	0.042	50.73	0.87	29.5
Ke T ½	hr	81.24	38.27	65.88	73.52	153.1	52.5
Ke	1/hr	0.01	51.71	0.01	59.13	0.0	45.3
MRT	hr	101.33	14.53	171.46	35.69	220.7	42.7
Tmax	hr	0.54	45.38	168.00	52.68	15.0	65.7
Tissue Penetration	%			44	51.93	910	44.6

AUC, Area-under-the-curve, from zero to the last time point (Cn), and from 0 to infinity; AUC % extrapolated is the percent extrapolated to infinity. Cmax, peak (maximum) concentration; Tmax, time to peak concentration; Penetration factor, ratio of AUC values of tissue site: plasma; Ke, elimination (terminal) rate, and associated half-life, T½; MRT, mean residence time.

## Discussion

A previous study from our group [11] showed that it is possible to measure active unbound drug concentrations in tissue fluids of enrofloxacin and it active metabolite, ciprofloxacin, in cattle injected with enrofloxacin at an approved label dose of 12.5 mg/kg. This study expanded upon previous work by measuring interstitial fluid concentrations, and concentrations at the site-of-infection (PELF) for other BRD therapeutic agents: florfenicol, ceftiofur, tulathromycin and enrofloxacin at a lower approved dose of (7.5 mg/kg). Importantly, this study also demonstrated a technique where calves could be sampled repeatedly in a cross-over study in a humane manner. Compared to other tissue studies, there was no need to sacrifice animals to collect tissues. At the end of the study, catheters and ultrafiltration probes were removed from the calves and after an appropriate withdrawal time were available for humane slaughter or transfer to another study.

In a review paper, Theuretzbacher [10] stated that, “*No studies that simultaneously employ various methods for the determination of antimicrobial concentrations in the lower respiratory tract or that are useful for comparing different body fluids such as PELF and ISF are available*”. This study corrects that deficiency, at least for drugs used for treating BRD. This was the first to measure antibiotic concentrations at both sites, in addition to the total concentration in plasma.

In addition to the pharmacokinetic analysis, we also used plasma protein binding to determine its influence on the distribution of each drug (or drug metabolite for ceftiofur). A cross-over study design was used to minimize inter-animal variations in drug concentrations and reduce the total cost of the study. The outcomes for each drug can be used to better understand the relationship between plasma unbound (protein unbound) drug concentrations, interstitial fluid (unbound) concentrations, and concentrations at the site-of-infection, the epithelial lining fluid. These results will have an important influence on our understanding of site of infection drug disposition and activity in cattle.

The importance of ISF is already known. Experts conclude that measurement of active drug concentrations in the extracellular fluid was the preferred method to correlate pharmacokinetic-pharmacodynamic (PK-PD) indices to clinical efficacy [8, 18]. Liu et al [8] concluded that antibiotic concentrations in the interstitial fluid are responsible of the antibacterial effect at the target site and are more relevant in predicting therapeutic efficacy than plasma concentrations. These and other experts [9] emphasized the importance of evaluating unbound drug concentrations, not total concentrations, for evaluating antimicrobial activity.

An FDA guidance has stated that pharmacodynamic studies should include relating the concentrations at the site of action to the in vitro susceptibility of the target microorganisms [19]. Therefore, this study was designed to expand our previous knowledge of ISF tissue fluid concentrations to evaluate concentrations in the PELF. Antibiotic concentrations in interstitial fluid can be predictive of the active concentration necessary for treating most infections. However, the respiratory tract presents another challenge: the diffusion of antibiotics across the blood-alveolar barrier (also referred to as the blood-bronchus barrier in some publications). The concentration of drug that penetrates the blood-alveolar barrier can be assessed by collecting the PELF. The PELF may be an important site of infection in pneumonia [20], but there are limitations on the interpretation of these data [7]. The importance of the drug concentration in the PELF for BRD in predicting efficacy was reviewed by Giguère & Tessman [1]. These authors concluded that for some drugs, “measurement of drug concentrations in pulmonary epithelial lining fluid is a better predictor of efficacy than either lung or plasma concentrations …”. However, there is not complete agreement with this assumption. The importance of adequate antibiotic concentrations in PELF notwithstanding, we also recognize that lung infection can disrupt the alveolar wall and invade the interstitial space. Therefore, a healthy pulmonary epithelium may not represent the actual environment during clinical infection [7]. In addition, during established pneumonia which may occur in BRD, the area of consolidation may not resemble PELF. In reality, both ISF and PELF concentrations may be important to evaluate: ISF drug concentrations may be predictive of respiratory concentrations during infection when disruption of the blood alveolar barrier occurs; but drug concentrations in PELF may be more predictive of drug concentrations in the airway secretions and may be helpful for infecting agents that colonize the airways. The results of this study may have important implications in determining the appropriateness of various antibiotics for treating BRD. It is possible that drugs that attain adequate concentrations in PELF (adequate to meet PK-PD targets) are appropriate to prevent colonization in the airway. When the airway is inflamed and the blood-alveolar (blood-bronchus) barrier disrupted, the infection can spread to the interstitium where the ISF fluid concentration is relevant.

Drusano [21] discussed the properties that make an antibiotic acceptable for treating pneumonia. Among these are the potency of the drug (low MIC values), adequacy of penetration to the PELF, and the concentration of free (unbound) drug in the interstitial fluid. Therefore, an ideal drug would therefore possess the properties of favorable potency, high lipophilicity, and low plasma protein binding.

The ISF concentrations for ceftiofur were significantly lower than those in a study by Halstead et al [4] in which they demonstrated much higher penetration into tissue cages after a ceftiofur sodium dose of either 2.2 or 4.4 mg/kg. The measured penetration in that study (calculated from a ratio of the AUC values) ranged from a mean of 47% to 79%, depending on the dose and frequency of administration. This difference can be explained by the method of sample collection. Tissue cages collect fluid containing protein and other transudate components. Drug concentrations in transudate represent both the bound and unbound form. Our study collected protein-free ultrafiltrate, which represents the biologically active form. Penetration into the PELF, on the other hand, is close to the degree of penetration reported in the study by Halstead et al [4] who used a method of collecting PELF that was almost identical to our method. They reported penetration of 30.5% and 42.2% at the high and low dose, respectively. Although our calculation of *fu* (0.067) predicted the penetration of the ceftiofur metabolite into the ISF, the penetration into the PELF (from both studies) was higher than predicted the fraction of drug unbound (*fu)* for the ceftiofur metabolite (Table 2). Thus, there may be other factors that are responsible for diffusion of the ceftiofur metabolite across the intact pulmonary epithelium. In the review by Kiem & Schentag [7] only three comparisons between drug concentration in PELF and plasma were available for β-lactam antibiotics. For each study, the ratio of PELF:plasma for these β-lactams was < 0.5.

The enrofloxacin and ciprofloxacin penetration from plasma to the tissue fluid (ISF) was similar to predictions and consistent with other studies. But it is unclear what factors caused lower concentrations in PELF. The concentrations of enrofloxacin and ciprofloxacin in PELF were 24% and 40%, respectively of the plasma concentration. However, this appears to be in agreement with a previous study in which enrofloxacin was administered to calves at a dose of 2.5 mg/kg [5]. In that study the authors point out that collection of bronchial secretions in calves with their technique was difficult and there were not enough samples for pharmacokinetic analysis. However, estimating the bronchial fluid concentrations from their figures (Figure 1 in their paper) it appears that peak (Cmax) PELF concentrations were 24% and 45% of the enrofloxacin and ciprofloxacin concentrations, respectively. Their study did not report AUC for the bronchial fluids. In a follow-up study we examined enrofloxacin and ciprofloxacin concentrations in PELF after administration of 12.5 mg/kg to calves and the same technique used in this study. (Results not shown, publication is in preparation.) At the higher dose, we observed the drug concentration in the PELF to be 124% of the plasma concentration. Therefore, there are obviously differences among studies, the cause of which are undetermined, but it may be related to differences among groups of calves, variability in the sampling method, or because of the higher dose administered in our most recent study.

The pharmacokinetics of tulathromycin presented in Table 7 can be compared to other published values and summarized in the review by Villarino et al, [22] (refer to Table 1 of the Villarino paper). In other studies, the terminal half-life was (mean) 90 and 64 hours in beef calves and Holstein calves, respectively. In this study we calculated a half-life of 81 hours (CV 38%). The reported plasma area-under-the-curve from zero to infinity (AUC) in Villarino’s paper was listed as 18.7, 14.1, and 14.0 μg hr/mL, depending on the study. In our study we found 14.48 (CV 28.61%). Thus, we believe based on these comparisons that our study was consistent with others with respect to plasma concentrations.

There were large differences shown between our study and a previous study [23] when comparing PELF tulathromycin concentrations. The PELF AUC to plasma AUC ratio in the study by Cox et al. [23] was 53 and in our study was 9.1. The PELF half-life for tulathromycin in Holstein calves was 330 hours [23] and in our study it was 153 hours (CV 52.5%). The peak (Cmax) and AUC in PELF were reported as 3,730 ng/mL and 492 μg hr/mL [23] and in our study was 867 ng/mL (CV 29.5%), and 87.6 μg hr/mL (CV 21.74%), respectively. We calculated the AUC from samples out to 288 hours and the Cox study calculated to 360 hours [23]. But this is probably not enough to account for the large differences in PELF concentrations between the two studies.

The differences in PELF concentrations between our study and the Cox study [23] can be attributed to several factors (see Table 2 in Villarino’s paper [22]). PELF concentration measurements are inherently highly variable and affected by the method of collection. The Cox et al study, as well as others listed in the Villarino review [24] collected samples for PELF measurement by bronchoalveolar lavage (BAL). This method flushes the entire airway, including alveolar space. The direct sampling method used in our study (direct microsampling technique) samples the bronchial fluid directly. The alveolar tissue is more richly perfused with blood and there is faster equilibrium between blood and the PELF. The bronchial mucosa receives less blood and is slower to equilibrate. This may partially explain the lower concentrations measured in our study.

In the review by Kiem & Schentag [7], the authors cited other examples in which the direct microsampling technique produced consistently lower antimicrobial drug concentrations than PELF collected via BAL method. They concluded that the direct microsampling method *“may offer an overall better correlation with microbiological outcomes”*. As mentioned above, we have conducted follow-up studies to measure PELF concentrations after injections of antibiotics in another group of calves. (Results not shown; publication is in preparation.) In our follow-up study we compared PELF with the BAL method and found that, for fluoroquinolones, the BAL technique consistently produced concentrations (based on AUC) that are 4–5 x higher than the PELF direct sampling method, and approximately 6 x higher at individual sampling points.

The advantages and concerns with this collection method versus BAL have been previously discussed [1, 25, 26]. These authors discuss the methodological considerations, the dilution factor caused by flushing large amounts of fluid in the airway, and the problems with using the “urea correction method” to adjust for this dilution. On the other hand, the direct swab technique described by Menge et al. [3] and Halstead et al. [4] does not dilute the sample and requires no correction. This method was validated by Yamazaki et al. [6], who provided evidence that this is a more reliable measure of the concentration in the PELF.

It is also described in the Villarino review that the BAL collections are subject to overestimation of drug concentration because of contamination. In a pig study in which tulathromycin was measured via bronchial sampling vs BAL, the authors concluded that, “*An overestimation of drug concentrations in PELF is strongly associated with the BAL fluid technique and*, *at least for tulathromycin in cattle*, *might have a relevant impact on PELF concentrations after 11 hr post administration of the drug*” [24]. Thus, we acknowledge the differences in these techniques, and between studies, and we believe that the direct sampling technique as performed in this study is the most valid technique to measure drug concentrations in the airways.

The discrepancies in sampling techniques notwithstanding, the concentrations measured in this study can be viewed in relation to the reported MIC values for cattle pathogens. As reported by the drug sponsor (Zoetis Animal Health, Florham Park, NJ, USA) BRD pathogens have tulathromycin MIC_90_ values that range from 1 to 4 μg/mL, with *Histophilus somni* the highest at 4 μg/mL and *Pasteurella multocida* the lowest at 1 μg/mL. At no point, in the PELF as measured in our study, was the mean concentration above the lowest MIC_90_ (1.0 μg/mL). In fact, only 1 out of the 6 calves had PELF concentrations slightly above this value. This raises questions about the property of tulathromycin that produces clinical benefits in treated animals. Perhaps, as suggested in other reviews, the anti-inflammatory effects of macrolides are responsible for clinical effects [27]. Tulathromycin was shown to have significant effects on neutrophils and inflammatory cytokines [28]. Obviously, additional study is needed to characterize the effects of tulathromycin and other macrolides in animals with pneumonia as this drug is widely used with perceived efficacy at the dose used in this study.

From this study there are differences among drugs in the penetration of antimicrobials into the ISF and PELF that may affect the therapeutic use. It appears that some antimicrobials are best for control of respiratory disease based on high PELF concentrations while others may be more effective for treatment of pneumonia due to high ISF concentrations. In Table 2, we presented the penetration values for ISF and PELF in relation to the drug’s protein binding (shown as fraction unbound) and lipophilicity (shown as LogD). For all drugs (or drug metabolite for ceftiofur) there is a relationship between protein binding and the penetration to the ISF; but, a similar relationship is not present for PELF. Likewise, lipophilicity (the higher the LogD value, the higher the lipophilicity) does not appear to influence the penetration into either ISF or PELF. Thus, the properties of protein binding and lipophilicity—often cited as determinants of drug penetration into the PELF—did not have an influence on the penetration of the antimicrobials administered to the calves of this study.

Limitations to this study are that we used a relatively small number of calves (n = 6 for each administration) and all the calves were from the same source and on identical diets and housing conditions. Other groups of calves of different breeds and conditions may produce other results. Importantly, these calves were all healthy. Clearly, studies are needed in animals with respiratory disease to determine the effect on pharmacokinetics and drug distribution into the sites of infection.

Antimicrobials from different classes have distinct differences in the penetration into both the ISF and PELF that do not always directly correlate with protein binding or lipophilicity. Drugs including florfenicol and ceftiofur with high PELF concentrations are expected to be effective in the control of respiratory disease while those with high ISF concentrations including enrofloxacin and florfenicol may be more effective in treatment of active respiratory infections.

## Supporting Information

S1 Fig Animation of Collection of PELF. (MP4)
