# Correction: Comparison of Active Drug Concentrations in the Pulmonary Epithelial Lining Fluid and Interstitial Fluid of Calves Injected with Enrofloxacin, Florfenicol, Ceftiofur, or Tulathromycin

**DOI:** 10.1371/journal.pone.0159219

**Published:** 2016-07-08

**Authors:** Derek M. Foster, Luke G. Martin, Mark G. Papich

There are errors in the values in the “PELF” column, sub-column “Mean” for rows “AUC (0 to infinity)” and “AUC (0 to Cn).” Please see the corrected Table 7 here.

**Table 7:** Plasma, Insterstitial Fluid (ISF), and Pulmonary Epithelial Lining Fluid (PELF) Pharmacokinetics after Administration of 2.5 mg/kg of Tulathromycin to Calves.

		Plasma		ISF		PELF	
Parameter	Units	Mean	CV%	Mean	CV%	Mean	CV%
AUC % extrapolated	%	8.25	37.96	15.88	86.29	24.9	54.1
AUC (0 to infinity)	hr*μg/mL	14.48	28.61	8.60	7.50	119.90	27.6
AUC (0 to Cn)	hr*μg/mL	13.23	26.62	5.54	39.75	87.64	21.7
Cmax	μg/mL	1.82	82.28	0.042	50.73	0.87	29.5
Ke T ½	hr	81.24	38.27	65.88	73.52	153.1	52.5
Ke	1/hr	0.01	51.71	0.01	59.13	0.0	45.3
MRT	hr	101.33	14.53	171.46	35.69	220.7	42.7
Tmax	hr	0.54	45.38	168.00	52.68	15.0	65.7
Tissue Penetration	%			44	51.93	910	44.6

AUC, Area-under-the-curve, from zero to the last time point (Cn), and from 0 to infinity; AUC % extrapolated is the percent extrapolated to infinity. Cmax, peak (maximum) concentration; Tmax, time to peak concentration; Penetration factor, ratio of AUC values of tissue site: plasma; Ke, elimination (terminal) rate, and associated half-life, T½; MRT, mean residence time.
